# Publisher Correction: Mnemonic prediction errors bias hippocampal states

**DOI:** 10.1038/s41467-020-19656-2

**Published:** 2020-11-09

**Authors:** Oded Bein, Katherine Duncan, Lila Davachi

**Affiliations:** 1 Department of Psychology, New York University, New York, NY 10003 USA; 2 Department of Psychology, University of Toronto, Toronto, ON M5S 3G3 Canada; 3 Department of Psychology, Columbia University, New York, NY 10027 USA; 4 Center for Biomedical Imaging and Neuromodulation, The Nathan S. Kline Institute for Psychiatric Research, Orangeburg, NY 10962 USA

**Keywords:** Cognitive neuroscience, Hippocampus, Long-term memory, Human behaviour

Correction to: *Nature Communications* 10.1038/s41467-020-17287-1, published online 10 July 2020.

The original version of this Article contained an error in Fig. 2 panels b and c, where the asterisks representing significance were inadvertently shifted slightly to the left. The significance asterisks should have directly aligned only with the bars labelled “2” on the *x*-axes. This has been corrected in both the PDF and HTML versions of the Article.

