# Supplementary material for: Mnemonic prediction errors bias hippocampal states
Source: Nat Commun. 2020 Jul 10;11:3451. doi: 10.1038/s41467-020-17287-1 (PMC7351776; doi:10.1038/s41467-020-17287-1)
Supplement: Supplementary file 1 — Supplementary Information [file 41467_2020_17287_MOESM1_ESM.pdf]

Supplementary Information

Mnemonic prediction errors bias hippocampal states

Bein, O., Duncan, K. and Davachi, L.

<u>This supplemental includes:</u>

Supplementary Figures 1 and 2

Supplementary Notes 1-6

Supplementary Figures

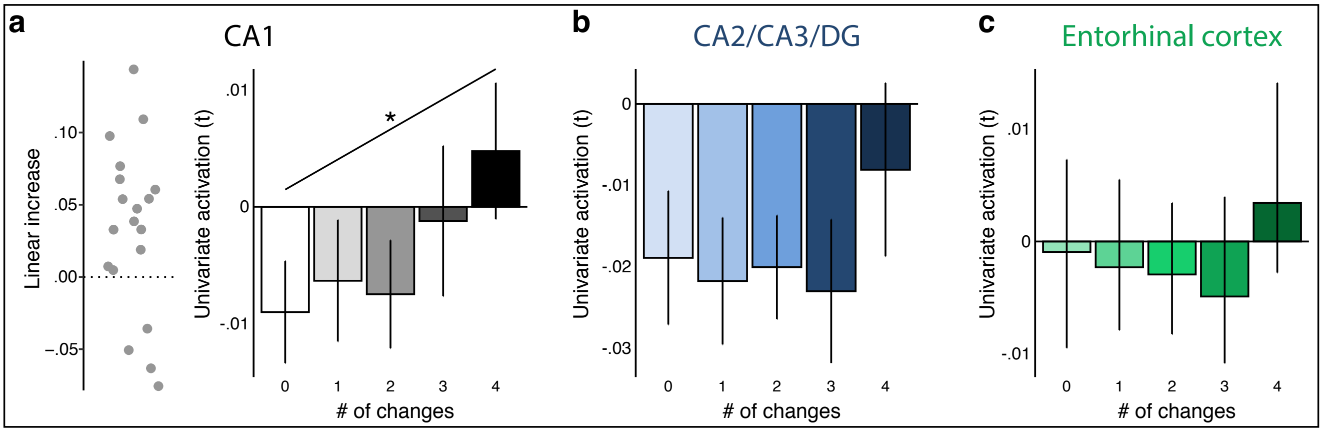

Supplementary Figure 1. Univariate activation in **a.** CA1, **b.** CA2/CA3/DG and **c.** entorhinal cortex. In CA1 (a), * p = .026, the results reflect a linear trend contrast across number of changes, tested against 0 with a one-sample, two-tailed, t-test (see main text). The dots in the dots plot reflect individual participants' linear trend contrast score. All data are from the left hemisphere (see main text). # of changes: number of changes. N = 19. Data in the bar graphs are presented as mean values, error bars reflect +/- SEM. Source data are provided as a Source Data File

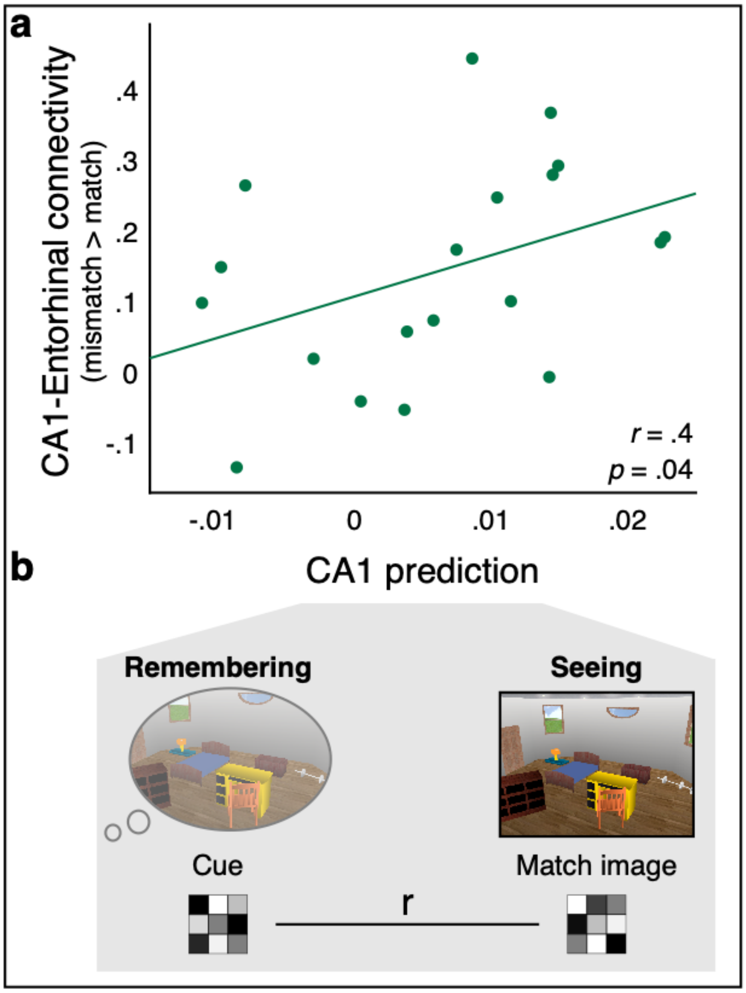

Supplementary Figure 2. **a.** CA1 prediction strength correlated with increase in functional connectivity between CA1 and Entorhinal cortex. As a connectivity measure, we took the mismatch - match contrast score for each participant (see main text). **b.** We quantified prediction strength by computing multivariate representational similarity between the cue part of a trial, and the match image of the same room (see main text for controlling for average "room" prediction by subtracting the similarity to match images of other rooms). N = 19. "r" denotes Pearson's correlation coefficient, p value is the result of a one-tailed t-test for a Pearson correlation. Detailed methods and results are provided in Supplementary Note 1. Source data are provided as a Source Data File.

Supplementary Notes

Supplementary Note 1 (related to Supplementary Fig. 2): Functional connectivity between CA1 and entorhinal cortex correlates with mnemonic prediction strength. In the main text we reported room-specific reinstatement in the left CA1, suggesting memory based-predictions in CA1 during the cue part of the trial. Here, we asked whether functional connectivity between CA1 and entorhinal cortex or between CA1 and CA3 were modulated by the strength with which an individual uses the cue to internally generate the memory-based prediction. While participants were all extensively trained on all 30 rooms in the experiment, we could ask whether variance in mnemonic reinstatement across individuals correlates with CA1 connectivity in response to room alterations.

To that end, we took for each participant the similarity of the cues to their corresponding matching images, and subtracted the average similarity of the cue to match images corresponding to other rooms (i.e., the room-specific reinstatement, see main text, Methods and Results). As a measure of CA1-entothinal cortex connectivity for each participant, we took the match < mismatch contrast score (0-changes vs. all levels of changes) because this score best characterized increases in CA1-entorhinal connectivity when viewing altered rooms in our experiment. Following the same logic, we used the linear contrast score as a measure of CA1-CA3 decreases in connectivity, since this contrast accounted better for CA1-CA3 connectivity. Thus, note that we are correlating two within-participant measures: (1) The similarity of the cue to its own matching room, compared to the similarity of the cue to other rooms (i.e., we are computing room-specific reinstatement, and subtract from that "baseline" similarity of the cue to rooms more broadly). (2) The increase (or decrease) in connectivity in response to changes in the images, namely, the match < mismatch contrast score (in CA1-entorhinal cortex connectivity) or the linear decrease contrast (CA1-CA3) per participant. This more conservative approach to across participants correlation ensures that we are not correlating two baseline and potentially trait-level brain measures, but rather a within-participant measure of the strength of room-specific reinstatement, with the increase in connectivity in response to errors. We tested for significance of the correlation using a one-tailed t-test for Pearson's correlation. The computational models that motivated our work[3,4]

provide strong theoretical foundations for a directional hypothesis. Specifically, they argue that more novelty - and specifically, stronger mnemonic prediction errors, should lead to a shift towards an encoding 'state' mediated by CA1-entorhinal connectivity. Thus, we hypothesized that prediction errors should specifically increase CA1-entorhinal connectivity while also decreasing CA1-CA3 connectivity - both are directional hypotheses and warrant one-tailed tests.

We found a significant correlation between prediction-strength in CA1 and the increase in CA1-entorhinal connectivity (*Pearson's r* =.40, CI: [.012-.68][1], *p* = .044, one-tailed; Supplementary Fig. 2), lending further support for the notion that functional connectivity increases are related to predictions and their violations. Prediction strength did not correlate with CA1-CA3 decreases in connectivity (linear decrease score, better accounting for connectivity changes between CA1 and CA3: *r* = .16, *p* = .26; match > mismatch score: *r* = .23, *p* = .17; one-tailed).

Although the results of increased functional connectivity between CA1 and entorhinal cortex in response to errors, and CA1 prediction, each separately holds when controlling for univariate activation (see Results, main text, and Supplementary Note 3 below), we sought to further control for univariate activation specifically for the correlation itself. To that aim, we conducted a multiple regression analysis whereby connectivity was the explained variable (the match < mismatch contrast, as in the main correlation), and as explaining variables we included the reinstatement measure, cue and intact image univariate activation, as well as the match < mismatch contrast of both CA1 and entorhinal cortex univariate activation. The reinstatement measure significantly explained variance in connectivity, even when controlling for these 4 additional variables (standardized $beta_{(reinstatement)}$ = .41, CI: [.03-.79], $t_{(13)}$ = 1.93, *p* = .039). We then repeated this analysis, but using a robust regression analysis (as implemented in R, using the rlm function), and obtained similar results (standardized $beta_{(reinstatement)}$ = .42, CI: [.04-.79], $t_{(13)}$ = 1.83, *p* = .045). These results demonstrate that the correlation of reinstatement (i.e., prediction strength) with connectivity is unlikely to be driven by univariate activity.

---

[1] Confidence interval for the correlation, as well as for the coefficients reported in the multiple regression control analyses, were computed at a 90% confidence, in line with the directional hypothesis.

Supplementary Note 2: Controlling for accuracy and reaction times in CA1 connectivity.

In the main paper, we tested whether theoretically driven contrasts, namely, a linear contrast and a match > mismatch contrast, can characterize the patterns of CA1 connectivity changes. However, one might wonder whether other more data-driven variables, such as accuracy rates or reaction time (RT) in the different conditions, might account for the connectivity data better than our theoretically driven contrasts. This might be a concern especially in CA1-CA3 connectivity, whereby the decrease in connectivity might parallel the decrease in reaction times.

To directly test the idea that differences in accuracy rates or RTs across participants or levels of changes may account for our connectivity findings, we conducted a mixed-level models that included accuracy and RTs, and our results hold. Specifically, for each pair of regions (CA1-entorhinal/CA1-CA3), we included connectivity as the explained variable, and accuracy rates and RTs per participant and per each level of changes as explaining variables. In line with the contrasts that explained more variance in connectivity (see main text), in CA1-entorhinal, we added the match < mismatch contrast as our primary explaining variable, and in CA1-CA3, we added the linear contrast our primary explaining variable. We then compared these full models to models that included only the behavioral factors as explaining variables. Our model comparisons showed that for both pairs of regions, the full model that included the primary contrast of interest significantly outperformed the model that only included the behavioral data, without the contrast of interest (CA1-entorhinal: χ^2 = 10.29, p = .0013, AIC or BIC reductions > 5; CA1-CA3: χ^2 = 12.18, p = .0005, AIC or BIC reductions > 7). We further note that comparing the full model to a model including only the match < mismatch (for CA1-entorhinal) or the linear trend contrasts (for CA1-CA3) did not significantly explain more variance, suggesting that accuracy and RT do not explain variance in connectivity beyond the variance explained by level of changes (CA1-entorhinal: χ^2 = 0.03, p = .98, AIC or BIC were lower for the simpler match < mismatch model that did not include the behavioral factors, differences > 3; CA1-CA3: χ^2 = 1.13, p = .57, AIC or BIC were lower for the simpler model, including only the

linear model, differences > 2). The results did not change when conducting the same model comparisons but considering accuracy data and RTs separately.

Together, these analyses show that accuracy rates or reaction times are unlikely to account for the connectivity findings. Thus, while some aspects of medial-temporal lobe as well as parietal lobe activity definitely correspond to levels of confidence in mnemonic judgements[1,2], in our task such potential confidence levels, as reflected in accuracy and RT, did not influence functional connectivity.

Supplementary Note 3: Control for univariate activation in CA1 prediction strength analysis.

Our main analyses revealed a significant effect of room-specific reinstatement in the left CA1, namely, higher similarity between the cue and the intact (0-changes) image of the same room, compared to similarity of the cue to intact images of other rooms. To control for univariate activation, we computed, for each participant the average activation during the cues, and the match images we took for the analysis. Then, we included the reinstatement measure per participant as the explained variable in a multiple regression, and the average univariate activation per participant of the cues, and of the images. The intercept of the model thus corresponds to the magnitude of the reinstatement, when accounting for univariate activation. Indeed, the intercept was significant (intercept = .006, CI: [.0007-.011], $t_{(16)} = 2.41$, $p = .028$; note that this intercept is similar to the difference between same-room and other-rooms reinstatement in the main analysis, which was .0055, see Results, main text), suggesting that reinstatement cannot be explained by univariate activation.

Supplementary Note 4: Control for univariate activation in CA1 prediction error analysis.

We found that the representational similarity between the cue and the image was lower in left CA1 when changes were evident in the image, compared to when there was no change (match > mismatch contrast, namely, 0-changes vs. the average of all levels of changes). To control for univariate activation in the left CA1, we conducted a multiple regression model in which the prediction error match > mismatch contrast per participant was the explained variable. As explaining variables, we included the match > mismatch contrast score (namely, 0-changes vs.

the average of all levels of changes), which we computed once for univariate activation in the left CA1 during the cue, and once for the univariate activation in the left CA1 during the image. Note that here, the intercept reflects the effect of interest, namely, whether the match > mismatch difference is significantly different from 0, when controlling for univariate activation. Indeed, the intercept was highly significant (intercept = .008, CI: [.003-.0143], $t_{(16)}$ = 3.46, p = .0032), suggesting that CA1 multivariate prediction error signal cannot be explained by univariate activation.

Supplementary Note 5: Control for similarity to other rooms in CA1 prediction error analysis. One possibility is that rather than reflecting room-specific representational difference, the reduction in similarity that we observed stems from a general difference in the representational similarity of cues and intact images, compared to the similarities of cues to images that contain changes. To examine this possibility, we conducted a control analysis, in which, for each cue, we first computed the average correlation of that same cue to all other images that are in the same type of trial (i.e., same task, and number of changes), but correspond to other rooms. We then subtracted this similarity (correlation) of the cue to other rooms from the similarity of that cue to its corresponding image, to obtain room-specific similarity measure. Obviously, the temporal difference between the cue and other trials is not identical to the temporal difference between the cue and the same image, which are within the same trial. However, we control for time already in our main analysis, since for all level of changes, the cue-image timing is identical. Thus, the main analysis controls for time between the cue and the image, while this analysis controls for room-specificity. To the extent that our similarity difference reflects room-specific representations, the match > mismatch contrast should still be significant, even when subtracting similarity to other trials. Indeed, like in our main analysis, the match > mismatch contrast was significant (contrast M: = .01, SD = .016, CI: [.002-.017], $t_{(18)}$ = 2.60, p = .018). Echoing the main analysis as well, the difference between 0-changes and 2- or 3-changes were significant as well (t's$_{(18)}$ > 2.50, p's < .05). This analysis shows that our representational differences likely reflect room-specific representations in the left CA1, that are reduced when an image does not match to a retrieved memory.

Supplementary Note 6: RSA results without CA1 voxel selection.

In the main analysis, we employed a voxel-selection procedure to increase SNR for the representational similarity analyses (see main text). Here, we computed again the prediction strength measure, namely, the similarity between the cue and the 0-changes image of its corresponding room versus 0-changes images of other rooms (see main text), but using all left CA1 voxels. Consistent with our main analysis, we found a numerically greater similarity of CA1 activity patterns during the cue with the corresponding room, compared to the other rooms (match: M = .004, SD = 0.01; other: M = .0004, SD = .005; $t_{(18)}$ = 1.3, p = .22). Again consistent with our main analysis, we found a significant positive correlation of prediction strength with the increase in CA1-entorhinal connectivity in response to violations (*Pearson's r* =.51, p = .013[2]). Prediction strength did not correlate with CA1-CA3 decreases in connectivity also when using all CA1 voxels (linear decrease score, better accounting for connectivity changes between CA1 and CA3: r = .16, p = .74; match > mismatch score: r = -.12, p = .68).

We additionally computed the prediction error RSA analysis, like in the main analysis, but using all left CA1 voxels. We found that the representational similarity between the cue and the image in the left CA1 was lower when changes occurred in the image, compared to no changes, and this difference approached significance (match > mismatch contrast: M: = .005, SD = .01, CI: [-.0001-.01], $t_{(18)}$ = 2.07, p = .053, two-tailed).

These results show that the pattern of results reported remain largely similar without voxel selection, but also suggest that our approach of voxel selection was useful in enhancing SNR[5–7].

Supplementary References

1. Brown, T. I., Rissman, J., Chow, T. E., Uncapher, M. R. & Wagner, A. D. Differential Medial Temporal Lobe and Parietal Cortical Contributions to Real-world Autobiographical Episodic and Autobiographical Semantic Memory. *Sci. Rep.* **8**, 1–14 (2018).
2. Hutchinson, J. B. *et al.* Functional Heterogeneity in Posterior Parietal Cortex Across

[2] One-tailed t-test was conducted for this correlation, as well as for the correlation with CA1-CA3 connectivity, consistent with the main analysis.

Attention and Episodic Memory Retrieval. 49–66 (2014). doi:10.1093/cercor/bhs278

3. Hasselmo, M. E., Bodelón, C. & Wyble, B. P. A Proposed Function for Hippocampal Theta Rhythm: Separate Phases of Encoding and Retrieval Enhance Reversal of Prior Learning. *Neural Comput.* **14**, 793–817 (2002).
4. Meeter, M., Murre, J. M. J. & Talamini, L. M. Mode shifting between storage and recall based on novelty detection in oscillating hippocampal circuits. *Hippocampus* **14**, 722–741 (2004).
5. Favila, S. E., Chanales, A. J. H. & Kuhl, B. A. Experience-dependent hippocampal pattern differentiation prevents interference during subsequent learning. *Nat. Commun.* **6**, 1–10 (2016).
6. Chanales, A. J. H., Oza, A., Favila, S. E. & Kuhl, B. A. Overlap among Spatial Memories Triggers Repulsion of Hippocampal Representations Article Overlap among Spatial Memories Triggers Repulsion of Hippocampal Representations. *Curr. Biol.* **27**, 2307–2317 (2017).
7. Dimsdale-Zucker, H. R. & Ranganath, C. Representational Similarity Analyses: A Practical Guide for Functional MRI Applications. *Handb. Behav. Neurosci.* **28**, 509–525 (2018).
