# Supplementary material for: Mnemonic prediction errors bias hippocampal states
Source: Nat Commun. 2020 Jul 10;11:3451. doi: 10.1038/s41467-020-17287-1 (PMC7351776; doi:10.1038/s41467-020-17287-1)
Supplement: Supplementary file 3 — Reporting Summary [file 41467_2020_17287_MOESM3_ESM.pdf]

nature research

Corresponding author(s): Oded Bein, Lila Davachi

Last updated by author(s): Jun 11, 2020

Reporting Summary

Nature Research wishes to improve the reproducibility of the work that we publish. This form provides structure for consistency and transparency in reporting. For further information on Nature Research policies, see Authors & Referees and the Editorial Policy Checklist.

Statistics

For all statistical analyses, confirm that the following items are present in the figure legend, table legend, main text, or Methods section.

n/a	Confirmed	
☐	☒	The exact sample size (n) for each experimental group/condition, given as a discrete number and unit of measurement
☐	☒	A statement on whether measurements were taken from distinct samples or whether the same sample was measured repeatedly
☐	☒	The statistical test(s) used AND whether they are one- or two-sided<br>*Only common tests should be described solely by name; describe more complex techniques in the Methods section.*
☐	☒	A description of all covariates tested
☐	☒	A description of any assumptions or corrections, such as tests of normality and adjustment for multiple comparisons
☐	☒	A full description of the statistical parameters including central tendency (e.g. means) or other basic estimates (e.g. regression coefficient) AND variation (e.g. standard deviation) or associated estimates of uncertainty (e.g. confidence intervals)
☐	☒	For null hypothesis testing, the test statistic (e.g. F, t, r) with confidence intervals, effect sizes, degrees of freedom and P value noted<br>*Give P values as exact values whenever suitable.*
☒	☐	For Bayesian analysis, information on the choice of priors and Markov chain Monte Carlo settings
☐	☒	For hierarchical and complex designs, identification of the appropriate level for tests and full reporting of outcomes
☐	☒	Estimates of effect sizes (e.g. Cohen's d, Pearson's r), indicating how they were calculated

Our web collection on statistics for biologists contains articles on many of the points above.

Software and code

Policy information about availability of computer code

Data collection	No software was used as this study is a re-analysis of an existing data set.
Data analysis	MATLAB R2018b (Mathworks); FSL 5.0.2.2 (FMRIB; Smith et al., 2004); R version 3.5.2 (R Core Team, 2018); Lme4 (Bates et al., 2014);

For manuscripts utilizing custom algorithms or software that are central to the research but not yet described in published literature, software must be made available to editors/reviewers. We strongly encourage code deposition in a community repository (e.g. GitHub). See the Nature Research guidelines for submitting code & software for further information.

Data

Policy information about availability of data

All manuscripts must include a data availability statement. This statement should provide the following information, where applicable:

- Accession codes, unique identifiers, or web links for publicly available datasets
- A list of figures that have associated raw data
- A description of any restrictions on data availability

Minimally processed data and single-trial t-statistic maps that support the findings of this study are available on https://osf.io/re2wd/. Additional data will be provided from the corresponding author upon a reasonable request.

Field-specific reporting

Please select the one below that is the best fit for your research. If you are not sure, read the appropriate sections before making your selection.

☒ Life sciences ☐ Behavioural & social sciences ☐ Ecological, evolutionary & environmental sciences

Life sciences study design

All studies must disclose on these points even when the disclosure is negative.

Sample size	In this manuscript, we re-analyzed an existing data set, thus the sample size was determined by the previously collected data (N=20, Duncan et al., 2012). We reasoned this should be sufficient since it is larger or similar to the sample size of previous studies in our lab addressing functional connectivity between hippocampal subregions as a function of hippocampal states (Duncan et al., 2014), as well as investigating representational similarity between memory retrieval and perceptual input in hippocampal subfields (Tompary et al., 2016).
Data exclusions	One participant was removed from the reported experiment and all analyses due to substantial signal dropout in the entorhinal cortex. Specifically, this participant had only 12 voxels in the left entorhinal cortex and 80 voxels in the right entorhinal cortex. For comparison, other participants had on average 234 voxels in the left entorhinal ROI (range: 127-344), comprising 84% (range: 44%-93%) of the anatomical left entorhinal cortex. In the right entorhinal ROI, participants averaged 255 (range: 165-337) voxels, which were 87% (59%-95%) of the anatomical right entorhinal cortex.
Replication	We do not offer a replication, this is a single fMRI study. Future research should aim to replicate our findings.
Randomization	Our study was a within-participant design, in the sense that we recruited one group of participants, and data in all experimental conditions were collected in all participants.
Blinding	Blinding is irrelevant, as we had one group of participants.

Reporting for specific materials, systems and methods

We require information from authors about some types of materials, experimental systems and methods used in many studies. Here, indicate whether each material, system or method listed is relevant to your study. If you are not sure if a list item applies to your research, read the appropriate section before selecting a response.

Materials & experimental systems

n/a	Involved in the study	
☒	☐	Antibodies
☒	☐	Eukaryotic cell lines
☒	☐	Palaeontology
☒	☐	Animals and other organisms
☐	☒	Human research participants
☒	☐	Clinical data

Methods

n/a	Involved in the study	
☒	☐	ChIP-seq
☒	☐	Flow cytometry
☐	☒	MRI-based neuroimaging

Human research participants

Policy information about studies involving human research participants

Population characteristics	Twenty right-handed and native English speakers, healthy individuals with normal or corrected to normal vision were included in the current study (Mean age: 25.4 years, range 21-34). Further information can be found in Duncan et al. (2012), where the results of univariate analyses of these data were previously published.
Recruitment	Since the data already existed, no new participants were recruited for this specific study. The sample for the original report was recruited from the NYU community using posters. Bias in the selection is unlikely, as this is a within-participant design. All measures were taken within each individual participant.
Ethics oversight	The Institutional Review Board at New York University

Note that full information on the approval of the study protocol must also be provided in the manuscript.

Magnetic resonance imaging

Experimental design

Design type	event-related
Design specifications	For each participant we had 10 scanning session (5 per task, see Methods; the analyses reported are collapsed across tasks). Each scan included 27 trials, to make a total of 270 trials. Here, analyses were focused on the total number of changes (0-4 total changes). The analysis was conducted on 30 trials in each of the 0 and 4 changes, 60 trials in the 1 and 3 changes conditions, and 90 trials in the 2 changes condition (across both tasks). One participant had 8 blocks, and another had 7. The minimal number of trials per condition was 24 and 21, correspondingly, still allowing a meaningful

analysis. Hence these participants were included in the analysis. On Each trial, he room's name appeared for 1.5 s, followed by 1 s blank and a probe image (4 s). A variable ITI followed the probe presentation and lasted between 1 and 13.5 s (Duncan et al., 2012).

Behavioral performance measures

Accuracy and reaction times were recorded, and means and standard deviations are reported in the manuscript in each number of changes (both collapsed across tasks, and in each task). The average accuracy was above 50% chance rate. We statistically examined the difference in accuracy rates and reaction times between the number of changes (0-4) using a repeated-measures ANOVA. As expected, participants were overall more accurate and quicker to respond in the 0-changes (presumably, an "easy" match response) and 4-changes (presumably, an "easy" mismatch response) trial types, compared to 1/2/3-changes, in which detection of a mismatch should be more difficult. This confirms that indeed, participants performed the task as expected.

Acquisition

Imaging type(s)

functional, structural

Field strength

3T

Sequence & imaging parameters

Functional data was collected using a high-resolution echo-planar pulse (EPI) sequence similar to that used in Olman et al. (2009). TR=2.500 s, TE =49, FOV = 192 X 96, 26 interleaved slices, distance factor of 20%, 1.5 X 1.5 X 2 mm voxel size, 10 runs, 125 volumes per run. The oblique coronal slices were aligned perpendicular to the hippocampal long axis (Duncan et al., 2012).

Area of acquisition

We did not have a full brain coverage. The area of acquisition was optimized to allow full cover the hippocampus and MTL with the limitations of the TR and the voxel size, and applying the oblique slices, as detailed above. This resulted in coverage of the hippocmapus and MTL regions for all participants. Typically, the FOV included other temporal and inferior frontal lobe regions, as well as parts of the brain stem.

Diffusion MRI ☐ Used ☒ Not used

Preprocessing

Preprocessing software

Preprocessing was done for the previous publication of the data using fsl and included standard motion correction with MCFLIRT and slice-timing correction (Duncan et al., 2012). A 3-mm smoothing was conducted masked for each ROI so that the smoothing is done within each ROI. The data was not preprocessed again for this study.

Normalization

All analyses were conducted within defined ROIs in the subject space, hence the data was not normalized to a standard space.

Normalization template

All analyses were conducted within defined ROIs in the subject space, hence the data was not normalized to a standard space.

Noise and artifact removal

Data was motion corrected (Duncan et al., 2012).

Volume censoring

The first 5 volumes were discarded to allow for signal normalization using FSL FEAT. No other volume censoring was performed. In the publicly available data, these volumes are already excluded.

Statistical modeling & inference

Model type and settings

To obtain the single-trial estimates we used an LSS (Least-Square-Separate) approach (Mumford, Davis, & Poldrack, 2014; Mumford, Turner, Ashby, & Poldrack, 2012; Turner, Mumford, Poldrack, & Ashby, 2012). Thus, in the first level analysis, a separate GLM was computed for each trial. Each model included the image portion of a single trial as a regressor of interest. The cue portion in all trials were included in one regressor. Other images were binned based on trial type to make 9 additional regressors of no interest. For the Representational Similarity Analysis we also estimated trial-level patterns during the cue period. Similarly to the image LSS models, a separate GLM was computed for each cue. Each model included the cue portion of a single trial as a regressor of interest. The other cues were included in one regressor. Images were binned based on trial type to make 9 additional regressors of no interest.

Effect(s) tested

Repeated measures ANOVA was conducted to test the effect of number of changes (0-4) on functional connectivity between the ROIs of interest (CA1-CA3/CA1-entorhinal). Mixed-level models (implemented by lme function in R) further characterized the observed functional connectivity, employing model-comparisons to determine the model that best characterize functional connectivity (using Chi-square test). Pearson's correlation was used to correlate participants' CA1-predictions, as evaluated by RSA analysis, with functional connectivity, followed by a t-test to determine significance. CA1 RSA mnemonic prediction error was estimated by computing a contrast per participant summarizing the RSA differences across number of changes (0-4) and comparing it to 0 using a one-sample t-test. Mixed-level models and multiple regressions were used to control for additional factors in supplementary analyses.

Specify type of analysis: ☐ Whole brain ☒ ROI-based ☐ Both

Anatomical location(s)

ROIs were manually drawn by KD on subjects' anatomical images, following previously established guidelines (Kirwan, Jones, Miller, & Stark, 2007; Insausti et al., 1998; Pruessner et al., 2002).

Statistic type for inference
(See Eklund et al. 2016)

Mean ROI values were taken to the analysis

Correction

We had an a-priori hypothesis on the ROI pairs

Models & analysis

n/a	Involved in the study
☐	☒ Functional and/or effective connectivity
☐	☐ Graph analysis
☐	☐ Multivariate modeling or predictive analysis

Functional and/or effective connectivity

Functional connectivity was was computed using a beta-series correlation approach (Rissman et al., 2004), in which a time series of single-trial activation values in two regions are correlated. As a measure of single-trial activation we used the t-statistic of trials resulting from the LSS models. These t-statistics were also used in our Representational Similarity analysis.

Graph analysis

Report the dependent variable and connectivity measure, specifying weighted graph or binarized graph, subject- or group-level, and the global and/or node summaries used (e.g. clustering coefficient, efficiency, etc.).

Multivariate modeling and predictive analysis

Specify independent variables, features extraction and dimension reduction, model, training and evaluation metrics.
